# Ionization Detail Parameters for DNA Damage Evaluation in Charged Particle Radiotherapy: Simulation Study Based on Cell Survival Database

**DOI:** 10.3390/ijms25105094

**Published:** 2024-05-07

**Authors:** Monika Mietelska, Marcin Pietrzak, Aleksandr Bancer, Antoni Ruciński, Zygmunt Szefliński, Beata Brzozowska

**Affiliations:** 1 Biomedical Physics Division, Institute of Experimental Physics, Faculty of Physics, University of Warsaw, 02-093 Warsaw, Poland; monika.mietelska@fuw.edu.pl; 2 Radiological Metrology and Biomedical Physics Division, Nuclear Facilities Operations Department, National Centre for Nuclear Research, 05-400 Świerk, Poland; marcin.pietrzak@ncbj.gov.pl (M.P.); aleksandr.bancer@ncbj.gov.pl (A.B.); 3 Laboratory of Translational Imaging in Oncology, Inserm, Institut Curie, Université Paris Saclay, 91401 Orsay, France; 4 Institute of Nuclear Physics PAS, 31-342 Krakow, Poland; antoni.rucinski@ifj.edu.pl; 5 Heavy Ion Laboratory, University of Warsaw, 02-093 Warsaw, Poland; szef@fuw.edu.pl

**Keywords:** nanodosimetry, track structure, Monte Carlo simulations, Geant4-DNA, radiation response, DSB, radiobiological cross sections

## Abstract

Details of excitation and ionization acts hide a description of the biological effects of charged particle traversal through living tissue. Nanodosimetry enables the introduction of novel quantities that characterize and quantify the particle track structure while also serving as a foundation for assessing biological effects based on this quantification. This presents an opportunity to enhance the planning of charged particle radiotherapy by taking into account the ionization detail. This work uses Monte Carlo simulations with Geant4-DNA code for a wide variety of charged particles and their radiation qualities to analyze the distribution of ionization cluster sizes within nanometer-scale volumes, similar to DNA diameter. By correlating these results with biological parameters extracted from the PIDE database for the V79 cell line, a novel parameter $R_{2}$ based on ionization details is proposed for the evaluation of radiation quality in terms of biological consequences, i.e., radiobiological cross section for inactivation. By incorporating the probability *p* of sub-lethal damage caused by a single ionization, we address limitations associated with the usually proposed nanodosimetric parameter $F_{k}$ for characterizing the biological effects of radiation. We show that the new parameter $R_{2}$ correlates well with radiobiological data and can be used to predict biological outcomes.

## 1. Introduction

Track structure analysis is gaining attention because it could examine the nature of radiation damage from a nanoscale perspective. However, a key challenge remains: bridging the gap between observed biological response and the underlying physical interactions at the nanometric scale. This motivates researchers in experimental nanodosimetry to develop new quantifiable measures that properly describe both aspects. Previous studies provide compelling evidence that differences in the effects of sparsely and densely ionizing radiation at the cellular and molecular levels can be explained by the complexity of local DNA lesions. Indeed, nearly three decades ago, it was postulated that the precise spatial arrangement of ionizing events along a particle’s trajectory through DNA significantly influences the complexity and repairability of double-strand breaks (DSBs) in irradiated cells [1].

Brenner and Ward [2] showed that the frequency of clusters of ionizations within a 2–3 nm target correlates well with the yield of DSB. A similar finding was soon reported by Michalik [3]. Also, the maximum relative biological effectiveness (RBE) for most biological endpoints occurs for 100 keV/µm of linear energy transfer (LET), which coincides with a ∼2 nm mean free path length for ionizations [1]. There is growing evidence that these observations are not just a coincidence but reflect the importance of clustered ionizations occurring at the scale of DNA diameter [4]. Therefore, the ionization cluster size distribution (ICSD) evaluation in nanometric volumes and nanodosimetric quantities derived from this basic concept are promising candidates for describing radiation quality [5,6].

The use of nanodosimetric quantities is of great importance for understanding the response of cells exposed to radiation that differs in the ionization density. With the ongoing development of nanodosimetric techniques supported with Monte Carlo (MC) simulations, it presents a valuable opportunity to improve the future planning of charged particle radiotherapy by considering ionization details. The evolving understanding of proton and ion radiobiology requires a more nuanced approach compared to current clinical methods relying on LET and RBE for charged particle radiotherapy; therefore, a formalism based on ionization detail parameters and cluster dose instead of current LET- and RBE-based models has been proposed for use in particle radiotherapy treatment planning [7]. For several years, researchers have been focusing on the nanodosimetric quantity expressing the probability of formation of the ionization clusters and its parameters for DNA damage evaluation. Descending to the nanometric scale allows us to count ionization acts in volumes comparable to short segments of DNA and link the number with DNA damage. For example, DSBs appear when two ionizations break two strands of a DNA molecule in close proximity. Based on the ionization pattern, the probability of occurrence of a given number of ionizations (i.e., a given cluster size)—aforementioned ICSD—can be calculated. ICSD is also used to estimate the cumulative probability $F_{k}$ of the formation of clusters of a given size (*k*) or larger. Representation of the ionization cluster size equal to or greater than two (described by cumulative probability $F_{2}$) as the yield of DSB of the DNA, at least, for electrons [8] is valid because of a similar dependence on energy.

In many studies, the potential use of $F_{k}$ was evaluated [6,7,9,10]. $F_{2}$ was chosen as the first quantity to assess because of the requirement for at least two ionizations leading to DSB. However, it became obvious that not every two ionizations lead to a double-strand break and hence the subsequent hypotheses about the validity of using $F_{3}$, $F_{4}$, or $F_{5}$. The index value selection in $F_{k}$ is not straightforward, but the question remains: is this choice even necessary? With $F_{k}$, regardless of the chosen *k* value, we consistently overlook events smaller than *k* and treat all other events as equally probable in causing a biological effect. Additionally, discrete values of *k* cause a discontinuous transition between $F_{k}$ and $F_{k+1}$, making it impossible to precisely fit biological outcomes. This effect has been noticed in studies where linear combinations of $F_{2}$ and $F_{3}$ were demonstrated, marking the first indication that perhaps clusters of sizes 2 and 3 should not be considered with the same probability [11]. The introduction of $R_{2}$, a new nanodosimetric quantity proposed in this work, addresses all these issues due to the continuous range of parameter values *p* along a biologically interpretable line and the intuitiveness of selecting a value in the index to be two because of the requirement for at least two ionizations leading to DSB.

Our studies aim to introduce a new descriptor for DNA damage after charged particle irradiation calculated based on ICSD parameters simulated with Geant4-DNA [12,13,14,15] and using experimental cell survival data. Being aware that a simple sum of probabilities associated with clusters of very different sizes may overestimate the assessment of the late biological outcome, based on previous findings and as a step toward novel dosimetry, we propose the use of the *R*_2_ quantity as a new approach in determining the biological effects of the exposure. In our approach, we decided not to assume that in each case, two ionizations are a sufficient condition to cause a DSB when considering a biological target. That is why we propose quantity $R_{2}$ based on binomial distribution and defined as:

(1) $$R_{2}\left(p\right)=\sum_{\nu =2}^{\infty }\sum_{k=2}^{\nu }P_{\nu }B_{k}\left(\nu ,p\right),$$

where $P_{\nu }$ is a probability distribution of cluster size ν, $B_{k}\left(\nu ,p\right)$ is the probability of *k* ionizations that have provided a sub-lethal lesion in a sequence of ν ionizations, and *p* is the probability of creating a sub-lethal lesion by a single ionization in this sequence. Here, a sub-lethal lesion is an elementary lesion that on its own is not harsh enough to drive apoptosis, but two or more of them within close range are enough to cause a DSB or severe lesion. The exact reasoning can be found in Section 4.1.2.

## 2. Results

### 2.1. Nanodosimetric Alternative Metric

**Figure 1:**
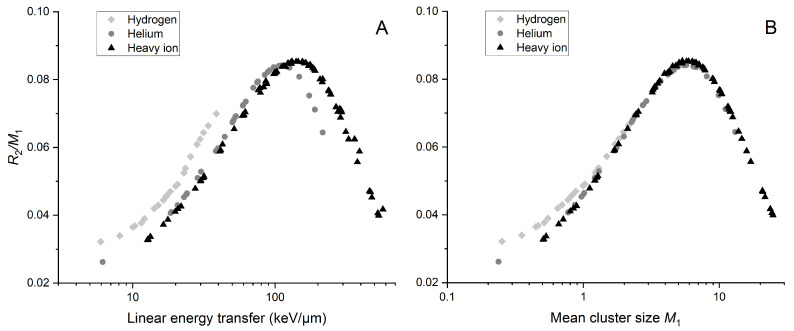
Ratio $R_{2}$ to mean cluster size $M_{1}$ as a function of linear energy transfer (**A**) and mean cluster size (**B**). $M_{1}$ values were simulated for a 2.3 × 3.4 nm^2^ target size. Data points are indicated by shades of gray and shapes representing ion type. “Hydrogen” includes protons and deuterons, while “Helium” includes He2+3 and He2+4 ions, along with alpha particles. Other used ions (^7^Li, ^11^B, ^12^C, ^14^N, ^16^O, ^20^Ne) are classified as heavy ions.

We use nanoscale quantities that describe local energy deposits to calculate $R_{2}/\;M_{1}$ rather than using dose values to calculate RBE, which we know are not the best descriptors of cellular response. Figure 1 shows $R_{2}/\;M_{1}$ as a function of LET (Figure 1A) and mean cluster size $M_{1}$ (Figure 1B). This reveals a characteristic curve resembling relative biological effectiveness as a function of linear energy transfer.

When plotting $R_{2}/\;M_{1}$ against LET, there is a significant shift of the maximum ratio toward lower LET when considering lighter ions. This effect is not noticeable when plotted against mean cluster size $M_{1}$. The maximum $R_{2}/\;M_{1}$ occurs at the LET and $M_{1}$ values for which we expect the maximum biological effectiveness (around 100 keV/µm of LET).

**Figure 2:**
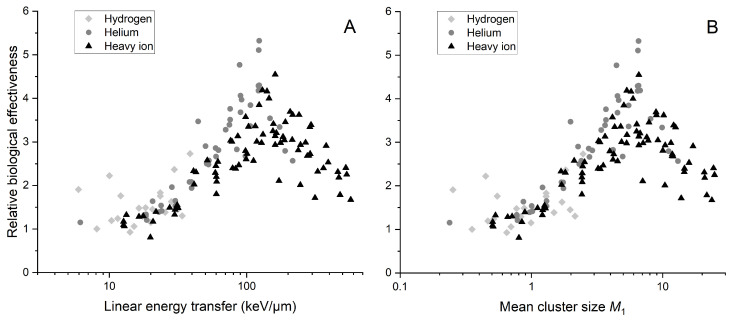
The RBE for a survival level of 5%, calculated as the ratio of dose from reference photon radiation to dose from ions, as a function of LET (**A**) and mean cluster size $M_{1}$ (**B**). The reference dose is computed using fitted survival parameters from the PIDE database for reference radiation, while the ion dose is determined using corrected survival parameters from PIDE for ion radiation. The simulation are performed for a 2.3 × 3.4 nm^2^ target size. Data points are differentiated by shades of gray and shapes representing ion type. The category “Hydrogen” includes protons and deuterons, while “Helium” includes He2+3 and He2+4 ions, along with alpha particles. Results for 8 experiments were not included due to insufficient information regarding reference radiation.

Consequently, we decided to calculate RBE for a 5% survival level using the α and β parameters of the linear-quadratic (LQ) model from the Particle Irradiation Data Ensemble (PIDE), which is presented in Figure 2. PIDE is a GSI biophysics project that compiles over 1200 pairs of in vitro cell survival experiment results after photon and ion irradiation from 131 publications. Data are stored in easily accessible file formats and include raw data, experiment specifications, linear-quadratic parameters and references. PIDE is continuously developed and it is freely available after registration. A disturbing observation in Figure 2 may be the dispersion of points in the graph. The reason for this difference may be that the experiments whose results were used to calculate RBE may have included studies that examined cells with different proportions in each phase. This is important because cell survival depends on the phase of the cell cycle. Another detail worth mentioning, but in this case much more specifically, is the presence of two outlier points for protons for low LET, exhibiting an RBE almost twice as high as the general trend. In the original paper, the reported α/β ratios for these points were about five times higher than five, usually reported for V79 cells. Given that this ratio is cell line specific, we could exclude these points. As this example shows, analyzing the dispersion would demand significant effort since many issues should be considered. PIDE does not provide uncertainties of presented values and certain other details regarding survival experiments that may lead to the exclusion of data from the analysis due to differences in experimental conditions. That is why we suggest considering this figure as a general trend indicating the advantages of using mean cluster size $M_{1}$, for which the RBE($M_{1}$) peak occurs for the same value of $M_{1}$ regardless of the ion type (except for protons) used to irradiate cells. Maximum RBE values for helium and heavy ions are obtained for different values of LET, 123.58 keV/µm and 161.32 keV/µm, respectively, but for the same value of $M_{1}$, approximately 6.6.

As can be seen in Figure 2A, the biological effectiveness may be higher for lighter than for heavier ions, even though they are described with the same LET value. As the ion’s mass number decreases, there is a pronounced shift of the RBE spectra towards the low-LET region. This relationship is not noticeable at the RBE graph plotted against mean cluster size $M_{1}$ (Figure 2B). When considering two different particles with the same LET, their biological effectiveness differs. However, two particles with the same $M_{1}$ exhibit the same biological effectiveness. In this context, $M_{1}$ appears to be a more suitable metric than LET for characterizing the quality of ionizing radiation. So, the trend presented in Figure 1 is also noticeable in this case.

### 2.2. Cell Inactivation Cross Sections

**Figure 3:**
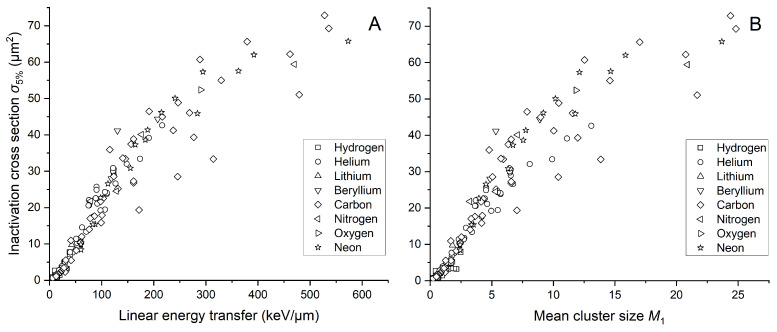
Inactivation cross section of the V79 cell line as a function of linear energy transfer (**A**) and simulated mean cluster size $M_{1}$ (**B**). LET was calculated using SRIM-2013 software based on energy from PIDE. Simulations are performed for a cylindrical target size of 2.3 × 3.4 nm^2^. The shape of each data point indicates the ion used for irradiating the cells. “Hydrogen” includes protons and deuterons, while “Helium” includes He2+3 and He2+4 ions, along with alpha particles. All other particles are considered heavy ions.

In response to the recognized limitations of linear energy transfer in fully characterizing radiation quality and its biological effects, we wanted to further explore the potential of $R_{2}$ as an alternative metric. However, this approach requires the calculation of another biological quantity. For this reason, we determined inactivation cross sections $\sigma_{5\%}$ for a 5% survival level and illustrated their relationship with both LET and mean cluster size $M_{1}$ in Figure 3A and Figure 3B, respectively. A 5% survival level was chosen because Belloni et al. [16] demonstrated that in this case, fitting parameters of the sigma dependence on LET indicate the same relationship regardless of the type of particle applied to irradiate the V79 cells. The mean cluster size was simulated based on the energy associated with LET for a cylindrical target with dimensions of 2.3 × 3.4 nm^2^ (diameter × height). Figure 3 visually demonstrates the obtained sigmoidal relationship. $\sigma_{5\%}$ increases until it shows a saturation effect for larger LET or $M_{1}$ values as a result of overkilling. We used various data point shapes to address data variance, especially regarding the ion used to irradiate the cells and study their survival. Despite noticeable fluctuations, we decided to use all data presented in Figure 3 for further analysis, acknowledging the particular biological variation. Additionally, we included all variants of the V79 cell line from the PIDE database. Notably, no significant differences were observed among the considered cell sub-types.

The mean cluster size $M_{1}$ use allows maintaining the sigmoidal shape of the dependency. The graph does not indicate deviations from the expected trend for any ion. Minor shifts in Figure 3B result from the fact that we used LET values to calculate $\sigma_{5\%}$.

### 2.3. Ionization Details Parameters

Based on the results of the nanodosimetric simulations, we analyzed the variation of $R_{2}$ as a function of mean cluster size $M_{1}$ for each tested target size *d* and the entire range of *p*. Observing that the curve shape corresponds to the previously identified pattern (see Figure 3) and using a direct proportionality between $F_{2}$ and $\sigma_{5\%}$ with the proportionality constant *K* established in prior studies [6], we aimed to identify the optimal parameters (*d*, *p* and *K*) that yield the best fit. This corresponds to minimizing the modified $\chi^{2}$ value as explained in Section 4.

**Figure 4:**
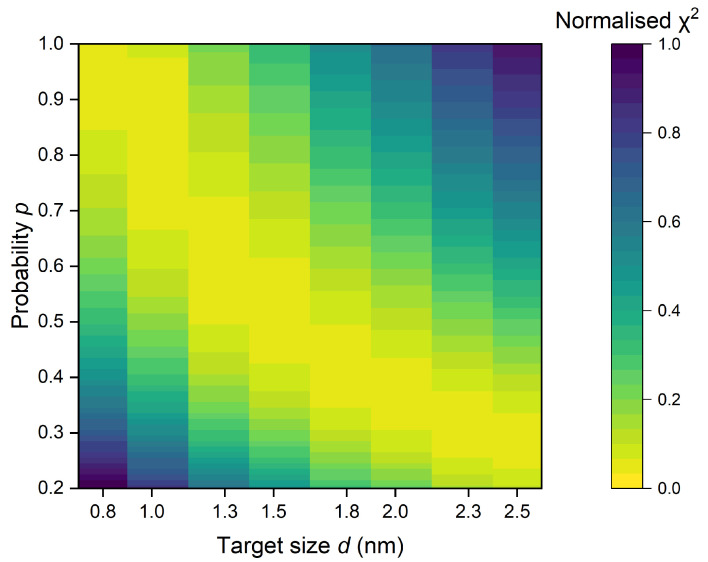
The heatmap, which displays the relationship between probability *p* and target size *d*, highlights the best fit between $\sigma_{5\%}$ and $R_{2}$ with *K* set to 57 µm^2^. Yellow areas indicate the region of optimal fit *d* and *p* parameters. $\chi^{2}$ is normalized to the highest $\chi^{2}$ value obtained within the specified range of *p* and *d*.

The heatmap, shown in Figure 4, illustrates the relationship between probability *p* of creating a sub-lethal lesion by a single ionization and target size *d*, depicting the best fitting between $\sigma_{5\%}$ and $R_{2}$ for *K* equal to 57 µm^2^. The variations observed are minimal, with the area of optimal fit (indicated by yellow) transitioning from small *p* and high *d* values to high *p* and low *d* values. While the heatmap provides valuable insights, it is important to note that identifying the best pair of *p* and *d* requires additional knowledge, such as the size of the DNA molecule.

We deduce that the consistency of $R_{2}$ with biological data for many *p* and *d* pairs is a result of the scaling procedure used to reach material equivalence [17]. Therefore, identifying the most accurate combination of *p* and *d* should involve focusing on the size of DNA fragments relevant from the ionizing radiation interactions perspective. This selection would lead to a *p* value, the assessment of which is more challenging. For this reason, these studies will focus on a target of 2.3 × 3.4 nm^2^ for which we obtained the best fit for p=0.35.

**Figure 5:**
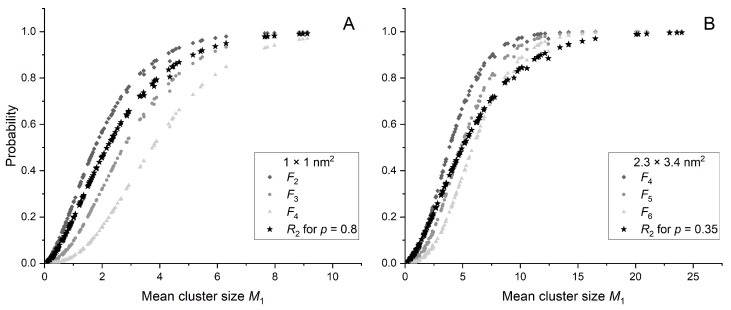
Probabilities $R_{2}$ and chosen $F_{k}$ as a function of $M_{1}$ for a target size of 1 × 1 nm^2^ (**A**) and 2.3 × 3.4 nm^2^ (**B**). Black stars represent the $R_{2}$ for *p* value corresponding to the best agreement with $\sigma_{5\%}$.

Figure 5 shows $R_{2}$ and a few selected cumulative probabilities $F_{k}$ as a function of mean cluster size for two different target sizes: 1 × 1 nm^2^ (Figure 5A) and 2.3 × 3.4 nm^2^ (Figure 5B). Cumulative probabilities $F_{k}$ were selected within the range of three consecutive *k* values for which the behavior of $F_{k}$ closely resembles that of $R_{2}$ as a function of mean cluster size $M_{1}$. For a target size of 1 × 1 nm^2^, this includes $F_{2}$, $F_{3}$, and $F_{4}$, while for 2.3 × 3.4 nm^2^, $F_{4}$, $F_{5}$, and $F_{6}$ are chosen.

The *p* parameter influences the $R_{2}$ curve slope as seen in Figure 5. The curve with *p* values closer to 1 (p=0.8 in Figure 5A) may pass between the curves $F_{k}$ and $F_{k+1}$. A significant decrease in *p* (p=0.35 in Figure 5B) allows the $R_{2}$ curve to intersect the curves $F_{k}$ and $F_{k+1}$. When using $F_{k}$, the discrete choice of curves makes fitting to biological data much more difficult.

### 2.4. Link between Radiobiology and Nanodosimetry

**Figure 6:**
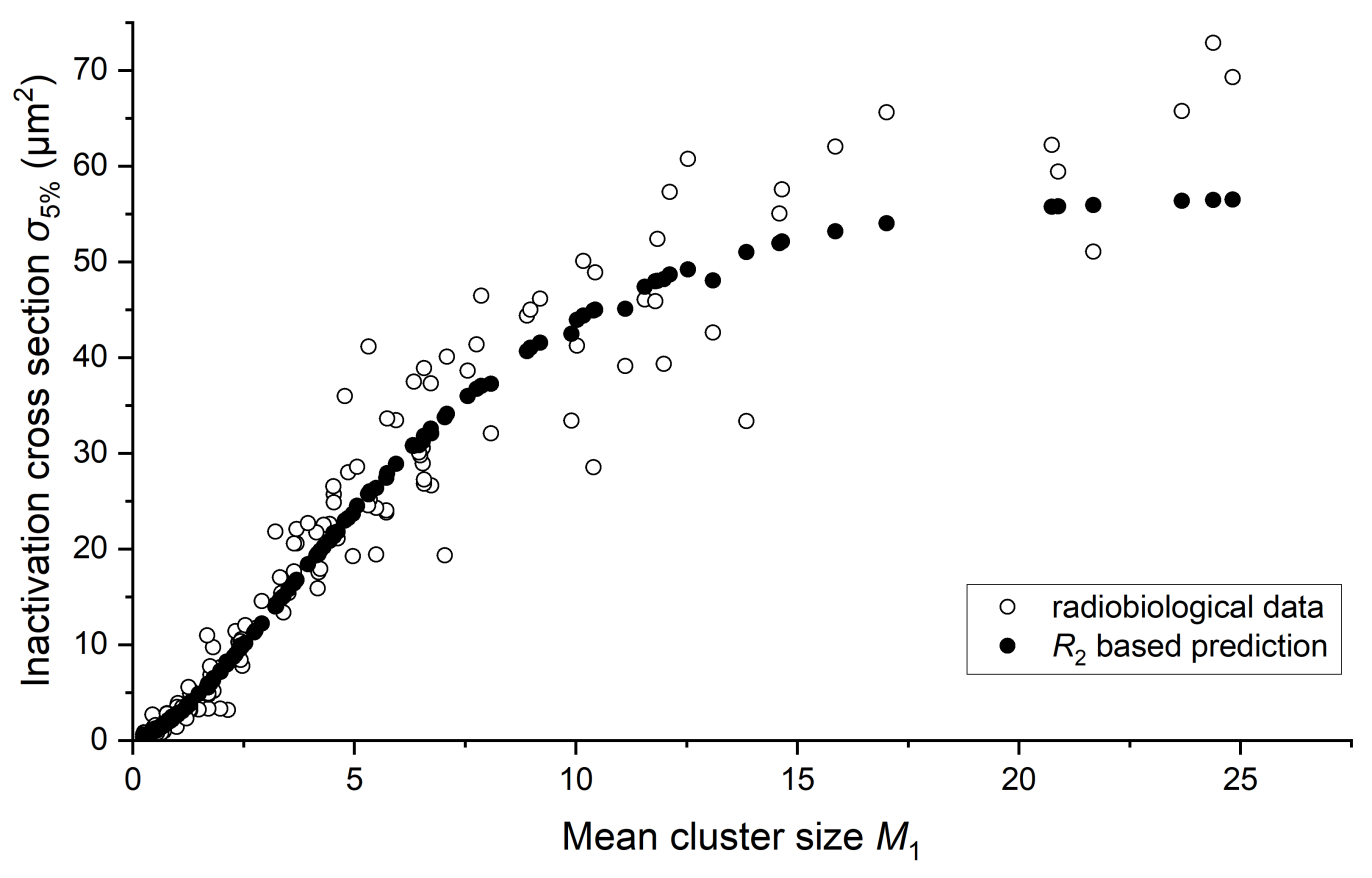
Inactivation cross section as a function of mean cluster size $M_{1}$, representing considered biological data, alongside the $R_{2}$ based model results represented by $R_{2}$ obtained for *p* equal to 0.35 and multiplied by the optimal *K* factor for a target size of 2.3 × 3.4 nm^2^.

Given the significant variability observed in the results across various target sizes *d* and probabilities *p*, which align well with biological data, we have chosen to focus on the findings regarding the 2.3 × 3.4 nm^2^ target size. This size represents short DNA segments, approximately one helical turn in length. We present $R_{2}$ values adjusted with the optimal *K* factor and compare them with $\sigma_{5\%}$ in Figure 6.

**Figure A1:**
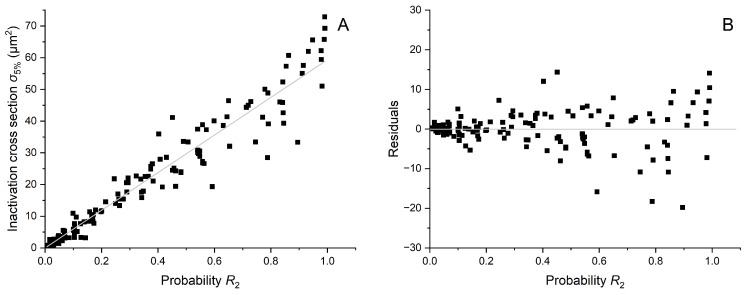
Linear fit between $\sigma_{5\%}$ and $R_{2}$ (**A**) and regular residuals of $\sigma_{5\%}$ in a function of an independent variable $R_{2}$ (**B**). Coefficient of determination equal to 0.971.

**Figure A2:**
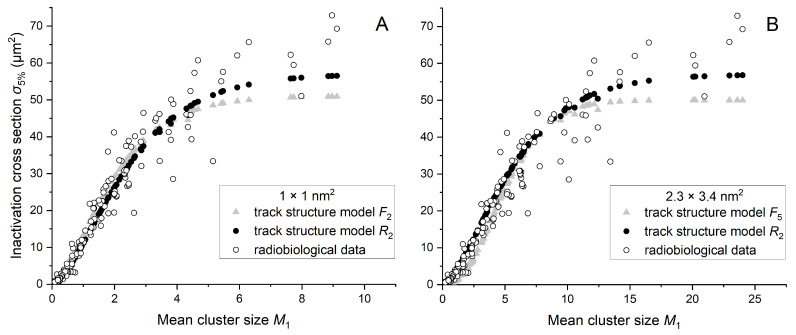
Comparison of $R_{2}$ and $F_{k}$ curves yielding the best fit with radiobiological data for target sizes of 1 × 1 nm^2^ (**A**) and 2.3 × 3.4 nm^2^ (**B**). In (**A**), the optimal fit is achieved with $F_{2}$ and $R_{2}$ for *p* equal to 0.8, while in (**B**), the best fit is observed for $F_{5}$ and $R_{2}$ for *p* equal to 0.35. In both cases, $R_{2}$ provides a better fit than $F_{k}$.

This demonstrates the relationship between the proposed nanodosimetric parameter $R_{2}$ and the biological metric across different mean cluster size $M_{1}$ values, revealing a strong alignment that suggests a significant correspondence. More details of this fit, particularly the justification for adopting simple proportionality between $\sigma_{5\%}$ and $R_{2}$ as well as a comparison with $F_{k}$, are presented in Appendix A. Figure A1 shows $\sigma_{5\%}$ plotted against $R_{2}$ for a specified target size (Figure A1A), along with the residuals of $\sigma_{5\%}$ plotted against $R_{2}$ (Figure A1B). Considering the possibility that $F_{k}$ might offer a better fit to radiobiological data compared to $R_{2}$, we present $F_{k}$, which yielded the best fit with radiobiological data, alongside $R_{2}$ for the *p* value that resulted in the best fit, shown as functions of $M_{1}$ in Figure A2.

**Figure A3:**
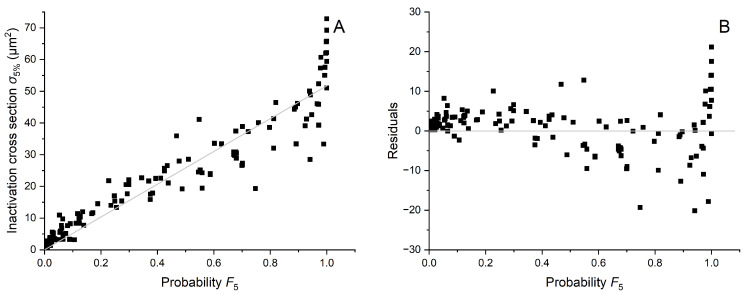
Linear fit between $\sigma_{5\%}$ and $F_{5}$ (**A**) and regular residuals of $\sigma_{5\%}$ in a function of an independent variable $F_{5}$ (**B**). Coefficient of determination equal to 0.955.

**Figure A4:**
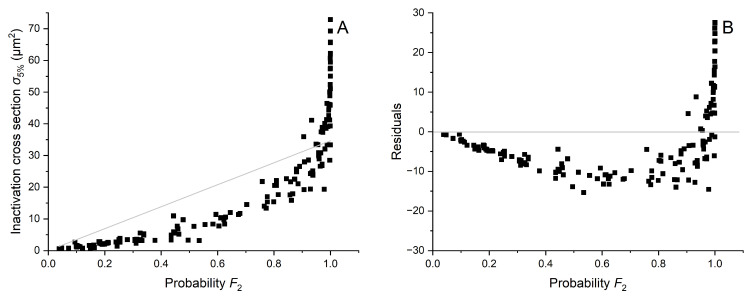
Linear fit between $\sigma_{5\%}$ and $F_{2}$ (**A**) and regular residuals of $\sigma_{5\%}$ in a function of an independent variable $F_{2}$ (**B**). Coefficient of determination equal to 0.839.

Additionally, to illustrate that $R_{2}$ correlates better with radiobiological data than with the discrete models provided by $F_{k}$, linear fit and regular residuals for $F_{5}$ and $F_{2}$ are calculated and presented in Appendix A as Figure A3 and Figure A4. $F_{5}$ was chosen as a $F_{k}$ providing best fit, while $F_{2}$ is a quantity including also events with two ionisations—the minimal number that can lead to DSB induction. The coefficient of determination calculated for all three models shows that the $R_{2}$-based model correlates the best (0.971), while for $F_{5}$ and $F_{2}$, it is equal to 0.955 and 0.839, respectively.

## 3. Discussion

The possibility of applying ionization detail parameters to describe the cellular response to ionizing radiation is supported by experimental cell survival data from the particle irradiation database [18]. The mean cluster size parameter, similar to LET, can be used to characterize particle track structure and its impact on the biological effect regardless of the ion type used for irradiation. The inactivation cross sections calculated using radiobiological data correlate well with the $R_{2}$ parameter based on the nanometric track structure. Both measured and simulated cross section are presented as a function of the mean cluster size instead of LET, which is the standard parameter for the interaction description at the macroscopic level. $R_{2}$ and $M_{1}$ are used, as they may be more suited to describe the stochastic nature of radiation and its effects, especially at the subcellular (nm) level and thus allow understanding of the cellular response to ionizing radiation in the context of DNA damage. The number of ionization acts in the volume of individual DNA nucleotides is the main factor determining the RBE of ionizing radiation.

Calculations based on the spatial distribution of the ionization acts can recreate the relation between RBE and LET (or $M_{1}$). Namely, for both RBE and $R_{2}\;/M_{1}$ plotted as functions of LET (or $M_{1}$), one can see the same shape of the curve, with the characteristic peak at about 100 keV/µm (or corresponding $M_{1}$ value). Ionization detail parameterization is closer to the phenomena description at the nanoscale than the average LET parameter. Both nanodosimetric measurements and Monte Carlo simulations allow estimating ionization detail parameters.

Based on ICSD parameters, we introduce $R_{2}$ as a novel metric describing the probability associated with DNA damage derived from the binomial distribution of the $P_{\nu }$. The subscript value of $R_{2}$ was selected based on the necessary condition for DSB (probability distributions of the induction of ionization clusters with a size equal to or greater than a fixed number). Unlike the discrete parameter *k* in $F_{k}$, $R_{2}$ uses the continuous parameter *p*, which may have a potential association with the chromatin arrangement within the nucleus of a given cell line. $R_{2}$ depends on the target size; however, due to biological variability, we could not determine significant differences among different target sizes. The chosen target size serves as a representative measure of biologically significant volume, equivalent to the short DNA segment, approximately one helical turn in length, or about 10 base pairs.

In previous studies [6,9], the best fit was achieved with *K* equal to 65 µm^2^, in contrary to presented here the value of 57 µm^2^, but it is important to note that the mentioned studies incorporated only selected biological data. Outliers below the $\sigma_{5\%}$(LET) curve were not considered, particularly those that underestimate *K*. In our study, inactivation cross sections were also derived from published in vitro survival parameters for the V79 cell line but we focus on a greater variety of ion types and radiation qualities. Additionally, we included all variants of the V79 cell line from the PIDE database. Notably, no significant differences were observed among the considered sub-types. In the cited publications, the quantity $F_{2}$ was used, and the best fit was obtained for 1 × 1 nm^2^. In subsequent studies [11] that employed a linear combination of $F_{2}$ and $F_{3}$ for a spherical volume with a diameter of 1 nm, this *K* value was found to be equal to 50 µm^2^ for the V79 cell line. Still, only selected data points were used, but the comparison is challenging due to the different shapes of the considered target. For this reason, we will temporarily focus on analyzing the results that correlated with $F_{2}$, which is a representation of $R_{2}$ with p=1. We deduce that the consistency of $F_{2}$ for a 1 × 1 nm^2^ target size with biological data is a simple result of the scaling procedure shown by [17]. A broad spectrum of probability *p* and target size *d* pairs yields acceptably good fits between nanodosimetric cumulative probability and biological data. This range begins with *p* approximately equal to 1 ($F_{2}$) and a target size of 1 × 1 nm^2^, extending as *p* decreases and *d* increases. Therefore, finding the most accurate *p* and *d* combination requires at least a general knowledge of the size of DNA fragments important in the context of interactions with ionizing radiation and a sensible approach to assessing the resulting *p* value based on the chosen *d*. For that reason, these studies focus on $R_{2}$ for a target of 2.3 × 3.4 nm^2^. Basing on $R_{2}$ is not contrary to the approach proposing ionization details [7]. It presents the opportunity to enhance this approach using the proposed universal descriptor $R_{2}$.

Another interesting feature of $R_{2}$ is the fact that it can be directly measured, as the ion counting efficiency η of a nanodosimeter can be tuned to the same value as the probability *p* in $R_{2}\left(p\right)$. In measurement practice, calculating $F_{2}$ from the ion cluster size spectrum obtained with a device characterized by ion counting efficiency η=p is equivalent to calculating $R_{2}\left(p\right)$ on the true spectrum of ionization clusters. This equivalence can be also interpreted by treating a DNA segment as a nanodosimeter with efficiency *p* being its ion detection efficiency. After all, a nanodosimeter is a physical model of the short DNA segment and its interaction with ionizing particles. In both systems (DNA and nanodosimeter), there is a certain number of ionizations and a detector that detects them either by being damaged (in the case of DNA) or by producing electric signals (in the case of nanodosimeter). If their detection efficiency is the same, the probability distribution of the number of DNA lesions in a DNA segment is the same as the measured ICSD. The possibility of the direct measurement of a biologically relevant nanodosimetric parameter is the key idea that drives the development of a small device that could be easily and routinely used in the clinical environment. The recent advancements in the thick-GEM technology [19,20] suggest that such a device can be proposed in a few years.

The concept of $R_{2}$ as a radiation quality factor that should replace $F_{k}$ is similar to the earlier attempts to predict DNA strand break yield [21]. Authors of the mentioned work also consider simple binomial distribution to calculate probability per projectile of generating a specific complex lesion, e.g., DSB. However, their model accounts also for the fact that to produce a DSB, at least one break on each strand should be considered. This nuance is neglected in $R_{2}$ presented in this work. As a result, $R_{2}$ can be directly measured using a standard nanodosimeter setup with a single target. Modeling the actual two-strand DNA in a nanodosimeter would require two neighboring targets in a close vicinity. Such experimental setups are not unthinkable and were in fact proposed recently [22,23,24]. However, the level of complication of these experiments prevents the use of such methods in a clinical environment, especially given that miniaturized nanodosimeters still struggle to provide even a single target model [19,20]. In conclusion, since we cannot define any radiation quality factor in isolation from the properties of the biological system, we should take at least this minimal step and account for the finite probability *p* of converting an ionization into a lesion. Making this minimal necessary step leads to the concept of $R_{2}$ that happens to be a measurable quantity well correlated to biological parameters like a radiobiological cross section for cell inactivation.

The beauty of the nanoscale approach to radiation therapy treatment planning is that the ionization detail parameter characterizes the radiation field regardless of the radiation type that created the cluster. In other words, the ID, frequency distribution of the ionization cluster size, or $R_{2}$ parameter are independent of the composition of primary particles in the radiation field. This paves the way for much easier and more precise radiation therapy treatment planning with various radiation quantities, for instance, multiple ions. The nanoscale approach based on ionization detail parameters is furthermore an opportunity for boron neutron capture radiation therapy [25], where low-energy lithium ions and alpha particles are the source of complex DNA lesion clusters that are lethal to cells. Further therapy enhancement methods, benefiting from boron [26,27,28,29,30] or gadolinium [31] radiation sensitizers exposed to a neutron radiation field produced by a proton beam in a patient, would greatly benefit from a unified nanoscale physics quantity that considers the radiation effectiveness of different particles in a stochastic way.

## 4. Materials and Methods

### 4.1. Nanodosimetric Data

#### 4.1.1. Jet Counter Nanodosimeter

Jet Counter (JC) is a fully functioning low-pressure gas-based detection system designed and developed in the National Centre for Nuclear Research, Poland. It allows creating an equivalent nanometric volume by injecting the target gas (such as N_2_ or C_3_H_3_) into one side of an open cylindrical space at a few millibars of pressure and registering ions being produced as a result of an incoming projectile. This cylindrical volume simulates short DNA segments of approximately one helical turn in length, equivalent to about 10 base pairs and 3.4 nanometers in length, adjustable by modifying gas pressure. Incoming ionizing particles directed through this gaseous nanometric target produce ions, which are then extracted, guided by electrodes, and quantified within a vacuum using an electron multiplier. The number of individual ionizations in a nanodosimetric target volume per energy deposition event is called the ionization cluster size ν. Using coincidence techniques such as counting ionizations alongside pulsed gases targeted simultaneously or by accumulating ionization counts over multiple coincidences, the system gathers the necessary data to prepare the ionization cluster size distribution spectra. The procedure of the density scaling application [17,32] is conducted to compare a measured number of ionizations produced in a gaseous sensitive volume with cluster sizes simulated with track structure simulations performed in water.

The work details of the JC device are extensively described in [33,34] for alpha particles, single electrons [34,35], and carbon ions [24,36,37]. In the middle of 2023, preliminary measurements were conducted with JC on a proton beam at the Institute of Nuclear Physics, Polish Academy of Sciences in Krakow, using the AIC-144 cyclotron. Experimental results obtained with Jet Counter were used as a validation of Monte Carlo codes as detailed in Appendix B.

#### 4.1.2. ICSD and Other Nanodosimetric Quantities

Nanodosimeters, such as JC, make it possible to measure the size distributions of ionization clusters that are formed as a result of a particle passing through a gaseous medium (ICSD) and to determine the average size of such a cluster. This quantity (often denoted $M_{1}$) is calculated as the first moment of ICSD using the following formula:

(2) $$M_{1}=\sum_{\nu =0}^{\infty }\nu P_{\nu }.$$

As the $M_{1}$ is the average number of ionizations produced in a nanometric volume (such as DNA), it is conceptually similar to restricted LET, as it takes into account ionizations caused primarily by low-energy electrons. However, as it is restricted geometrically and not energetically, it actually takes into account contributions from all secondary electrons but with different weights determined by the spatial distribution of ionization events produced by these electrons.

$F_{k}$ is one of the most strongly suggested nanodosimetric radiation quality parameters, as it is found to be proportional to radiobiological cross sections σ [6,9,10,38]. It is defined as the simple sum of probabilities of creating clusters of size *k*:

(3) $$F_{k}=\sum_{\nu =k}^{\infty }P_{\nu }.$$

**Figure 7:**
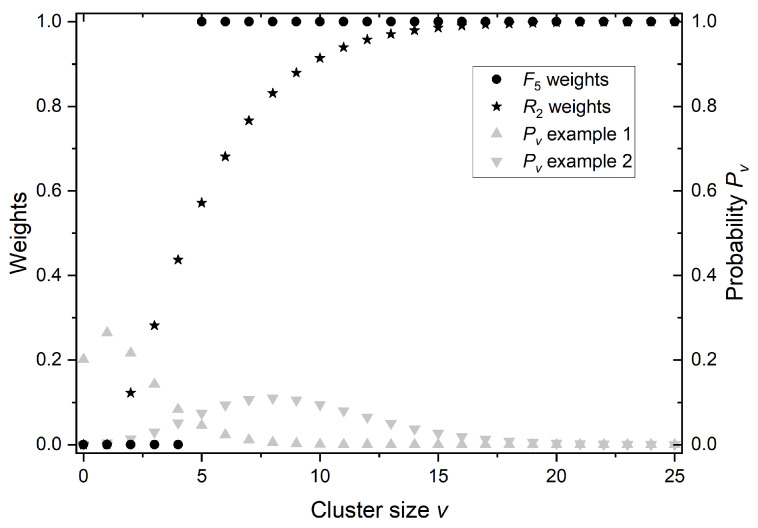
Comparison of weighting functions of $F_{5}$ and $R_{2}$ for p=0.35, along with two examples of ICSD ($P_{\nu }$).

This approach neglects all ionization clusters smaller than *k*. For example, $F_{3}$ takes into account events with 3 or more ionizations and does not include contributions of events with two ionizations. Taking into account only events strictly larger than 2 (k>2) is contradictory to the fact that two ionizations can be enough to cause a severe lesion like DSB. The purpose of this is to reflect the fact that larger clusters are more probable to cause a DSB. What it truly does is that it weights the probability $P_{\nu }$ of creating a cluster of size ν by a factor 0 for ν<k and factor 1 for ν≥k. However, a more natural approach is to assign a weight to each cluster size that reflects its probability of causing severe damage. In such case, clusters of size ν<2 should have a weight equal to 0 as they can, at best, create a single strand break (SSB). Clusters of size ν=2 should have the smallest but non-zero weight, and the larger the cluster is, the larger the weight should be, peaking at value 1 for large clusters, which most certainly will cause severe, irreparable damage. To quantify this idea, we propose considering the probability *p* that a single ionization creates a sub-lethal lesion, defined as a lesion that alone cannot lead to apoptosis but two or more of them in close vicinity are enough to do that. In such a case, the probability that a cluster of a given size will produce a lethal lesion is given by simple binomial distribution $B_{k}\left(\nu ,p\right)$. In conclusion, both $F_{k}$ and $R_{2}$ can be seen as a weighted sum of ICSD. The difference is that the weighting function of $R_{2}$ is not an arbitrarily chosen step function (as in the case of $F_{k}$) but a product of the stochastic nature of the considered phenomena. The difference is visualized in Figure 7 for weights of $F_{5}$ as an example, which is the best fit in Figure 5B among different $F_{k}$. For the same reason (best fit in Figure 5B), the value of *p*, for which $R_{2}$ weights are shown, was chosen to be equal to 0.35.

In Figure 7, two examples of ICSD are presented to illustrate how $R_{2}$ and $F_{5}$ weights interact with different ICSDs. ICSD of example 1 is composed mainly of small ionization clusters (low-LET radiation), while example 2 is composed mainly of large ionization clusters (high-LET radiation). In the case of low LET (example 1), $R_{2}=0.16$ and $F_{5}=0.09$. Such a small value of $F_{5}$ results from the complete neglect of clusters 2, 3, and 4, thus leading to the possible underestimation of the probability of severe lesions that those clusters may sometimes cause. On the other hand, in the case of high LET (example 2), $R_{2}=0.8$ and $F_{5}=0.9$, so the relative difference is small, but this time, $F_{5}$ may overestimate the probability of severe lesions. By the nature of $F_{k}$, choosing smaller *k* to compensate for the underestimation in the low-LET case would increase the overestimation in the high-LET case. In practice, i.e., in proton therapy, any chosen $F_{k}$ is going to underestimate the radiobiological effect in the healthy tissue and proximal part of a tumor (relatively low LET protons) and overestimate the effect in the distal part of the tumor and healthy tissue behind (relatively high LET protons).

#### 4.1.3. Monte Carlo Simulations

The accuracy and robustness of the used Monte Carlo models were first estimated by the comparison with nanodosimetric experiments presented in Appendix B. These simulations are based on the Jet Counter Monte Carlo simulation presented earlier for α particles [39] and recently extended to carbon ions [24]. This simulation code was built using the Geant4 (ver. 4.11.0.0) general-purpose MC simulation toolkit [40,41] with Geant4-DNA extension [12,13,14,15]. The Geant4-DNA option 4 [13,42,43,44] was used to simulate particle tracks in the sensitive volume (SV) of the detector down to the ionization threshold of around 10 eV. It contains models for elastic scattering, excitations, and ionizations in liquid water for all simulated particles. The processes were modeled in the track-structure mode simulating interactions in a step-by-step manner as opposed to the condensed-history mode applied in G4EmLivermore physics list. To simulate interactions in SV walls, the less accurate but faster models from G4EmLivermore list were applied with a 100 eV cut-off for ionization by electrons.

For the retrospective simulation representing radiobiological experiments reported in the PIDE database, only the Geant4-DNA option 4 was used, as it presents a good agreement with the nanodosimetric experiments. In these simulations, SV was a cylindrical nanometric target representing the short segment of DNA, which was placed in the center of 10 × 10 × 10 nm^3^ world filled with liquid water (G4_WATER material).

We completed simulations for target sizes of (1) diameters equal to height of 0.8, 1.0, 1.3, 1.5, 1.8, 2.0, 2.3 and 2.5 nm, (2) diameters of 1.0, 2.0, and 2.3 nm, and height/diameter ratio of 1.48. The source was a pencil beam of ^1^H, ^4^He, ^7^Li, ^11^B, ^12^C, ^14^N, ^16^O, ^28^Si to provide various radiation qualities and consistency with radiobiological data. Since Geant4-DNA can only simulate ionization caused by incident ions predominant in the cosmic spectrum, direct results for ^20^Ne were not available, leading to interpolation using ^12^C, ^14^N, ^16^O, and ^28^S. The interpolation method was tested for ^16^O using ^12^C, ^14^N, and ^28^S, yielding very good agreement with the result of the direct simulation for ^16^O. We used about 40 different energy points from 0.2 to 1000 MeV to evenly cover the whole energy range on a logarithmic scale. We obtained datasets with probabilities of creating a cluster size of different sizes for a given energy and LET calculated using SRIM software [45] for each tested particle type and target size. That allowed us to prepare ionization cluster size distributions (ICSDs) and calculate mean cluster size $M_{1}$ and cumulative probabilities $F_{k}$ of the formation of clusters sized equal to or greater than *k* for a given energy. These data enabled interpolation using a second-order polynomial to obtain $M_{1}$ for a given energy and particle used in radiobiological studies. We also used these ICSDs to calculate $R_{2}$ for a given $M_{1}$ and wide range of *p*. To keep the results at a similar level of uncertainties as in the nanodosimetric experiments with the Jet Counter, we simulated 100,000 particle histories for each case (particle type, energy, and target size).

Our final datasets for each target size and particle include energy (as mentioned), LET, $M_{1}$, $R_{2}$ for *p* from 0.2 to 1.0 ($F_{2}$) with a step of 0.01 and $F_{k}$ for k∈〈3,4,5,6,7〉. These datasets allowed us to interpolate all discussed quantities for energies and ions used in radiobiological experiments which we have chosen for further analysis.

### 4.2. Radiobiological Data

Radiobiological data were extracted from the particle irradiation data ensemble (PIDE) version 3.4 database [18,46]. Our focus lies on the linear (α) and quadratic (β) terms of the survival curves derived from graphs from many publications by the authors of PIDE. To obtain these parameters, they developed a computer program to digitize the data points in the figures. Subsequently, they determined α and β by fitting a second-order polynomial with an ordinary least squares fit to the negative logarithm of the survival.

We decided to focus our analysis on well-established asynchronous Chinese hamster lung fibroblast cells (V79), regardless of their sub-type, as we found no effect on the results obtained. Some points in the PIDE database are labeled as V79, while others are labeled as V79-4. We included all types since the category V79 includes both unspecified V79 cells and those with specific types (such as V79-379A, V79-754B, V79-S171, and even V79-4).

Survival parameters are driven from publications by many research groups around the world [47,48,49,50,51,52,53,54,55,56,57,58,59,60,61,62,63,64,65,66,67,68,69,70,71,72,73,74,75,76]. In these studies, we categorized experiments from PIDE using various criteria, such as ions used to irradiate cells. Under this classification, the “Hydrogen” category includes protons and deuterons, while “Helium” includes He2+3 and He2+4 ions, along with alpha particles from radiation sources. All remaining ions are classified as heavy ions. We narrowed studies to irradiation modalities labeled as monoenergetic and ions ranging from protons through neon Ne20, covering the LET range up to 528 keV/µm, which includes 152 survival curves. We used the LQ coefficients fitted by the authors of the PIDE database. In certain instances, the β parameter was fitted by them with a negative value, likely due to statistical fluctuations or systematic deviations. In such cases, we adopted the formalism provided by the authors of the PIDE database, adjusting the α parameter downward to compensate for β, thus obtaining an estimator for the best purely linear fit with β equal to 0. We did not use numerical values for the LQ coefficients from original papers to maintain consistency in our methodology.

The RBE was calculated as the ratio of the reference photon radiation dose to the dose required from tested ion radiation to achieve the same effect, specifically 5% survival. Doses were calculated by solving the equation $\alpha D+\beta D^{2}=-\ln\left(5\%\right)$ for *D*. The ion dose was determined using corrected survival parameters from PIDE for ion radiation, while the reference dose was computed using fitted survival parameters also from the PIDE database. The reference radiation included the following sources: ^60^Co, ^137^Cs, X-ray tube (100–300 kVp), or linear accelerator (6 MV).

Based on the α and β values from the PIDE database, we calculated the inactivation cross section $\sigma_{l}$ for a survival level of 5% for a monoenergetic ion beam, using the following equation [16]:

(4) $$\sigma_{5\%}=\frac{k}{\rho }\mathrm{LET}\sqrt{\alpha^{2}-4\beta \ln5\%},$$

where $\sigma_{5\%}$ is given in µm^2^, *k* is 0.1602 when LET is expressed in the unit of keV/µm, α in Gy^−1^ and β in Gy^−2^. We calculated LET based on energy from PIDE and using SRIM software. ρ is the density of the irradiated matter (≈1 g/cm^3^).

When $R_{2}$ values adjusted with the optimal *K* factor were compared with $\sigma_{5\%}$ to identify the optimal parameters yielding the best fit (*d*, *p* and *K*), we used the modified $\chi^{2}$, which is the sum of squares of differences between the value of σ for a given energy used in a given radiobiological experiment and $K\times R_{2}$ calculated for the respective energy corresponding to σ. This modified $\chi^{2}$ was calculated for all selected *K*, *p*, and *d*. For $R_{2}$, the optimal fit was achieved in all cases for K=57 µm^2^, regardless of varying the values or pairs of *p* and *d*. It can be assumed that the *K* factor represents a saturation threshold for a specific cell line and depends on its nucleus size.

## 5. Conclusions

Since the structure of the particle track is of great importance for the response of irradiated cells, our studies attempt to correlate the physical description of the radiation interaction with biological endpoints such as cell survival. In our work, we study nanodosimetric quantities in the context of their application to the interpretation of radiobiological data, as potential quantities that describe the complexity of biological damage. We conclude that the previously proposed cumulative probability overlooks a significant fraction of events in the target volume and treats other cluster sizes as evenly influencing the radiation response. This simplification contradicts the statistical character of ionizing radiation interactions with biological systems. Therefore, we propose considering a variable parameter *p* that reflects this complexity. These studies do not resolve the issue of finding the ultimate values of the *p* and *d* pair for a given cell line but indicate a general direction that should be explored so as not to lose the opportunity to use nanodosimetric description in the future charged particle radiotherapy planning. While a general formalism bridging the gap between nanoscale parameters and macroscale voxels used in the clinical practice of radiation therapy has recently been proposed, there is still a lack of knowledge and experimental data justifying the selection of appropriate ionization detail parameters to be applied. Our work on the PIDE database and proposal of the $R_{2}$ parameter represent important steps towards reaching a consensus. However, further validation studies, including in vitro and in vivo experiments, are required to conclusively determine the applicability of the nanoscale approach to radiation therapy treatment planning and radiation protection.

## Figures and Tables

**Figure A5:**
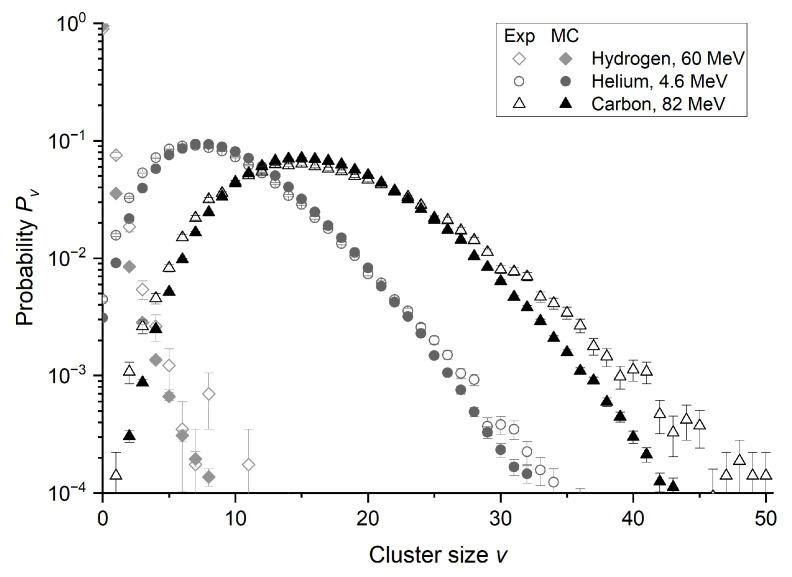
Comparison of ionization cluster size distributions obtained in experiments (Exp) with the Jet Counter device (empty shapes) and in Monte Carlo (MC) simulations (filled shapes) for hydrogen, helium, and carbon ions with different energies. All data are for a 2.3 × 3.4 nm^2^ target size. Uncertainties are due to statistical fluctuations.

## Data Availability

The data supporting the findings of this study are available from the corresponding author, B.B., upon reasonable request.

## Abbreviations

The following abbreviations are used in this manuscript:

DNA	Deoxyribonucleic Acid
DSB	Double Strand Break
GEM	Gas Electron Multiplier
GSI	GSI Helmholtz Centre for Heavy Ion Research, Darmstadt, Germany
ICSD	Ionization Cluster Size Distribution
JC	Jet Counter
LET	Linear energy transfer
LQ	Linear-Quadratic model
MC	Monte Carlo
PIDE	Particle Irradiation Data Ensemble
RBE	Relative Biological Effectiveness
SRIM	Stopping and Range of Ions in Matter
SSB	Single Strand Break

## Appendix A

To provide justification for the choice of a linear dependence between $\sigma_{5\%}$ and $R_{2}$, we illustrate in Figure A1 the relationship between $\sigma_{5\%}$ and $R_{2}$ with the best linear fit (shown in gray in Figure A1A), as well as the regular residuals of $\sigma_{5\%}$ with respect to $R_{2}$ as an independent variable in Figure A1B, both for a chosen target size of 2.3 × 3.4 nm^2^. Both graphs demonstrate that the linear dependence effectively captures the variability in the data, with approximately half of the points lying above and half below the best fit line, while also showing an expected increase in deviation as the $R_{2}$ values increase.

To illustrate that $R_{2}$ correlates better with radiobiological data than $F_{k}$, we present both $R_{2}$ and $F_{k}$ curves alongside radiobiological data as functions of $M_{1}$ for 2.3 × 3.4 nm^2^ (Figure A2B) and 1 × 1 nm^2^ (Figure A2A). In all cases, we selected $R_{2}$ for *p* that provided the best fit, and $F_{k}$ for *k* that resulted in the best fit (with *k* up to 7 tested). For the 2.3 × 3.4 nm^2^ target size, the best fit was obtained for $R_{2}$ with p=0.35 and $F_{5}$, while for the target of 1 × 1 nm^2^, it was achieved for $R_{2}$ with p=0.8 and $F_{2}$.

As shown in Figure A2, $F_{k}$ proves to be a suitable candidate only for a limited range of $M_{1}$ where we observe a linear dependence between $\sigma_{5\%}$ and $M_{1}$. However, at higher $M_{1}$ values where saturation occurs, $F_{k}$ no longer provides an ideal fit. Specifically, for higher $M_{1}$, $F_{k}$ tends to lie below the biological data. Adjusting the factor *K* could improve the model’s performance for $M_{1}$ values above the average, yet it tends to reduce the model’s accuracy, especially for average $M_{1}$ values at which the shift from a linear to a saturation phase happens. This crucial range is heavily represented with biological data points and substantially impacts the model’s fit. The discrete options provided by $F_{k}$ for choosing *k* index result in limited curve slopes, unlike the continuous parameter *p* selection in $R_{2}$, which allows for more precise fitting across a wide range of $M_{1}$ values, including within the saturation region.

To provide comparison with $F_{k}$ yielding best fit, Figure A3 shows the relationship between $\sigma_{5\%}$ and $F_{5}$ with the best linear fit (shown in gray in Figure A3A), as well as the regular residuals of $\sigma_{5\%}$ with respect to $F_{5}$ as an independent variable in Figure A3B, both for a chosen target size of 2.3 × 3.4 nm^2^. We also included similar results for $F_{2}$ in Figure A4 showing that $F_{2}$ for a given target size does not provide a satisfying fit.

## Appendix B

As the study relies on findings derived from Monte Carlo (MC) simulations, we aim to demonstrate the comparability between simulation outcomes for various particles and experimental results obtained using nanodosimeter. The comparison between ICSDs obtained using Geant4-DNA MC code and those measured with Jet Counter nanodosimeter (JC) is presented in Figure A5.

Details of the performed simulations and experiments can be found in [24,39]. The discrepancy between experimental data and simulations is more noticeable in the tails of the ionization cluster size distribution corresponding to large ionization clusters. This is primarily due to the increased experimental uncertainty of the Jet Counter and the reduced statistical uncertainty of simulations. The probability of large cluster sizes is relatively low, which is why the input for the cumulative probability does not cause significant differences.

The prediction of ICSD and their parameters based on Geant4-DNA simulations is satisfying and accurate enough to describe experimental data collected with Jet Counter for different charged particles (protons, alpha particles, and carbon ions) in a wide range of energies [24,39]. Our results indicate that the prediction of ICSD and their parameters based on Geant4-DNA Monte Carlo simulations is precise and opens the possibility of its application for other kinds and energies of the ionizing particles presented in this study.
